# Differentiating Unimodal and Multimodal Distributions in Pulsed Dipolar Spectroscopy Using Wavelet Transforms

**DOI:** 10.21203/rs.3.rs-3216615/v1

**Published:** 2023-08-04

**Authors:** Aritro Sinha Roy, Jack H. Freed, Madhur Srivastava

**Affiliations:** 1 Department of Chemistry and Chemical Biology, Cornell University, Baker Laboratory, Ithaca, 14853, NY, USA.; 2 National Biomedical Resource for Advanced ESR Spectroscopy, Cornell University, Baker Laboratory, Ithaca, 14853, NY, USA.; 3 Cornell Atkinson Center for Sustainability, Cornell University, 340 Tower Road, Ithaca, 14853, NY, USA.

**Keywords:** Electron spin resonance, Pulsed dipolar spectroscopy, Protein structure determination, Continuous wavelet transform, Time-frequency analysis

## Abstract

Site directed spin labeling has enabled protein structure determination using electron spin resonance (ESR) pulsed dipolar spectroscopy (PDS). Small details in a distance distribution can be key to understanding important protein structure-function relationships. A major challenge has been to differentiate unimodal and overlapped multimodal distance distributions. They often yield similar distributions and dipolar signals. Current model-free distance reconstruction techniques such as Srivastava-Freed Singular Value Decomposition (SF-SVD) and Tikhonov regularization can suppress these small features in uncertainty and/or error bounds, despite being present. In this work, we demonstrate that continuous wavelet transform (CWT) can distinguish PDS signals from unimodal and multimodal distance distributions. We show that periodicity in CWT representation reflects unimodal distributions, which is masked for multimodal cases. This work is meant as a precursor to a cross-validation technique, which could indicate the modality of the distance distribution.

## 1. Introduction

Protein structure determination remains one of the most challenging and open research subjects. Site-directed spin labeling (SDSL) [1–4] in combination with pulsed dipolar spectroscopy (PDS) has enabled protein structure determination using electron spin resonance (ESR) spectroscopy [5–10]. Typically, a pair of spin probes are attached to the domain of interest in a protein utilizing SDSL. The dipolar coupling between the spin probes at distance r apart is inversely proportional to $r^{3}$. Thus, measuring the dipolar coupling by PDS yields inter-spin distance information. Such distances can resolve aspects of protein structures directly and serve as crucial constraints in structural studies.

**Fig. 1:**
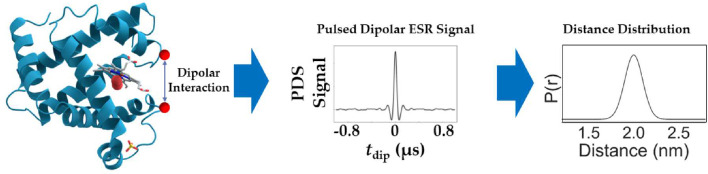
PDS signals capture the dipolar interaction between a pair of spin labels attached to a protein molecule. Post processing of the signal yields the distance distribution, P(r), between the spin pair.

Proteins are highly dynamic entities and their conformational ensembles give rise to distance distributions between the spin pairs, P(r), rather than a single value of r, as shown in Figure 1. The process of deriving P(r) from a PDS time domain signal, S(t), is an ill-posed problem [11, 12]. In general, a PDS signal can be expressed as

(1) $$S\left(t\right)=\int \;dr\kappa \left(t,\;r\right)P\left(r\right)$$

where κ(t,r) is the kernel that depends on t and r, averaged over the angle θ between the inter-spin dipolar vector and the direction of the external magnetic field. For all PDS techniques, κ(t,r) is singular and therefore, Equation 1 cannot be solved for P(r) by a simple inversion of κ(t,r). Various techniques have been proposed over the years to derive P(r) from PDS signals, including model-free [13–18], model-based [19–21] and training-based methods [22, 23]. In model-free approaches, such as Tikhonov regularization (TIKR) [13] and SF-SVD [16, 17], distance distributions are heavily reliant on the PDS time domain signals, as they operate independently of a priori information. Because of the nature of the problem of determining P(r), the solutions raise uncertainties, especially when the P(r) contains weak and/or shoulder peaks. In such cases, often there is no way to confirm whether the solutions truly represent the P(r) or they are artifact driven.

### 1.1. Major Challenge: Reconstruction of Small Details in P(r)

The kernel for DEER is given by

(2) $$\kappa (t,\;r)=\int_{0}^{1}\;cos\left[\left(1-3\;{cos}^{2}\theta \right)at/r^{3}\right]d\;cos\;\theta$$

where the dipolar constant, $a=\mu_{0}\mu_{B}^{2}g_{e}^{2}/2h,$ $\mu_{0}$ is the magnetic constant, $\mu_{B}$ the Bohr magneton, $g_{e}$ the free-electron g value and h the Planck constant. The DEER signal in its discrete form can be written as

(3) S=KP

where K and P are the kernel matrix and distance distribution vector. Note that the expression given in Equation 3 corresponds to the DEER signal originating from the interaction of an isolated pair of spin-1/2 particles. In a standard DEER experiment, the inter-molecular signal (or background) must be removed first.

**Fig. 2:**
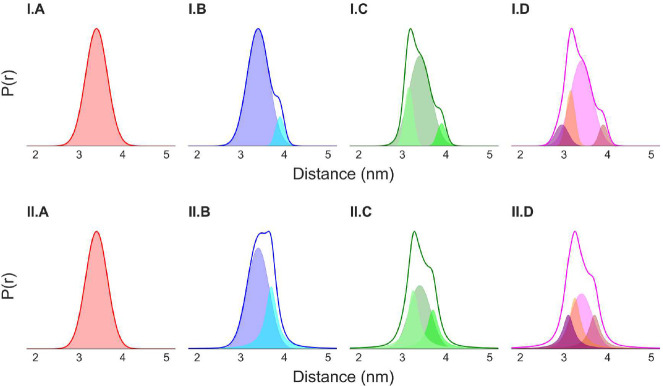
The different distance distributions, P(r), used in the analysis. The traces show the P(r) and the shaded regions show the different components of a distance distribution. Set-I P(r) were produced by mixing Gaussian distributions and set-II were produced by mixing Gaussian and Cauchy distributions with different means $\left(\mu_{r}\right)$, standard deviations $\left(\sigma_{r}\right)$ or width factor $\left(\Gamma_{r}\right)$ in different proportions (ϕ) : (I.A) $3.4\;nm\;\left(\mu_{r}\right)$ and $0.25\;nm\;\left(\sigma_{r}\right)$, (I.B) 3.4 nm and $3.9\;nm\;\left(\mu_{r}\right),$ 0.25 nm and $0.1\;nm\;\left(\sigma_{r}\right),$ 1.0 and 0.1(ϕ), (I.C) 3.15 nm, 3.4 nm and $3.9\;nm\;\left(\mu_{r}\right),$ 0.1 nm, 0.25 nm and $0.1\;nm\;\left(\sigma_{r}\right)$, 0.26, 1.0 and 0.1(ϕ) and (I.D) 2.95 nm, 3.15 nm, 3.4 nm and $3.9\;nm\;\left(\mu_{r}\right),$ 0.15 nm, 0.1 nm, 0.25 nm and $0.1\;nm\;\left(\sigma_{r}\right),$ 0.15, 0.26, 1.0 and 0.1(ϕ), (II.A) $3.4\;nm\;\left(\mu_{r}\right)$ and $0.25\;nm\;\left(\sigma_{r}\right),$ (II.B) 3.4 nm and $3.7\;nm\;\left(\mu_{r}\right),$ $0.25\;nm\;\left(\sigma_{r}\right)$ and $0.3\;nm\;\left(\Gamma_{r}\right),$ 1.0 and 0.01(ϕ), (II.C) 3.25 nm, 3.4 nm and $3.7\;nm\;\left(\mu_{r}\right),$ $0.3\;nm\;\left(\Gamma_{r}\right),$ $0.25\;nm\;\left(\sigma_{r}\right)$ and $0.3\;nm\;\left(\Gamma_{r}\right),$ 0.015, 1.0 and 0.01(ϕ) and (II.D) 3.1 nm, 3.25 nm, 3.4 nm and $3.7\;nm\;\left(\mu_{r}\right),$ $0.3\;nm\;\left(\Gamma_{r}\right),$ $0.3\;nm\;\left(\Gamma_{r}\right),$ $0.25\;nm\;\left(\sigma_{r}\right)$ and $0.3\;nm\;\left(\Gamma_{r}\right),$ 0.01, 0.015, 1.0 and 0.01(ϕ).

The P(r) used in the simulations are shown in Figure 2. All the distance distributions in model-I (top row) were produced by mixing different Gaussian distributions (shaded area). For model-II (bottom row), both Gaussian and Cauchy distributions (defined in Equation 4) were mixed in producing the distance distributions. The P(r) for each model is so designed that they are very similar with minor differences. Such small differences in the P(r) can be key in understanding protein structure-function relationships and structural changes.


(4) $$\text{Gaussian}\;\text{distribution}:f_{G}\left(r,\;\mu_{r},\;\sigma_{r}\right)=\frac{1}{\sigma_{r}\sqrt{2\pi }}e^{-{\left(r-\mu_{r}\right)}^{2}/2\sigma_{r}^{2}}\;\text{Cauchy}\;\text{distribution}:\;f_{C}\left(r,\;\mu_{r},\;{\text{Γ}}_{r}\right)=\frac{{\text{Γ}}_{r}}{{\left(r-\mu_{r}\right)}^{2}+{\text{Γ}}_{r}^{2}}$$

**Fig. 3:**
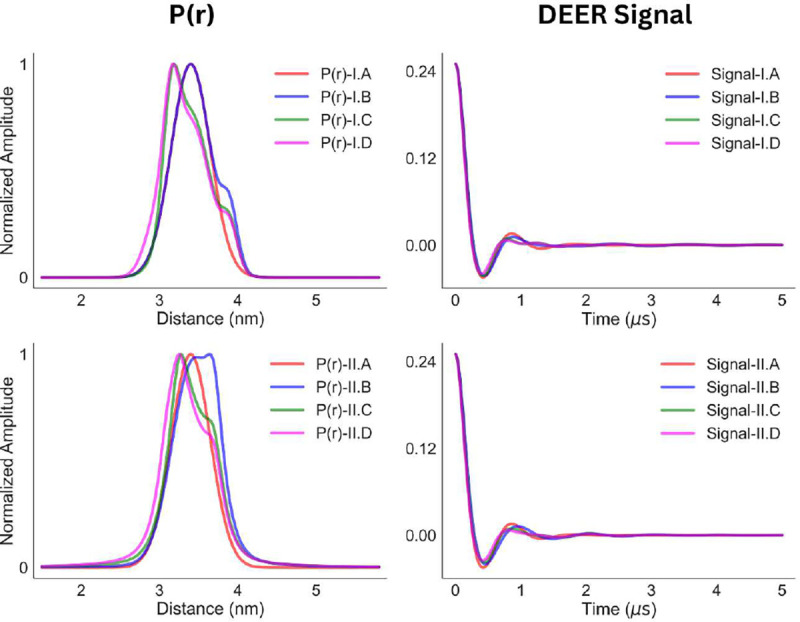
Two different sets of P(r) (left panel) and the corresponding DEER time traces (right panel) are shown. The DEER signals were calculated with dipolar evolution time of 5 *μ*s and a time increment of 6 ns.

It is often challenging to reconstruct such small details in the P(r) with great confidence. The DEER signals utilized for model-I and -II distance distributions are shown in Figure 3. Visual inspection of the DEER time traces hardly shows any differences, while the differences in their distance distributions are visible in the overlapped plots of the scaled P(r) (the left panel of Figure 3).

**Fig. 4:**
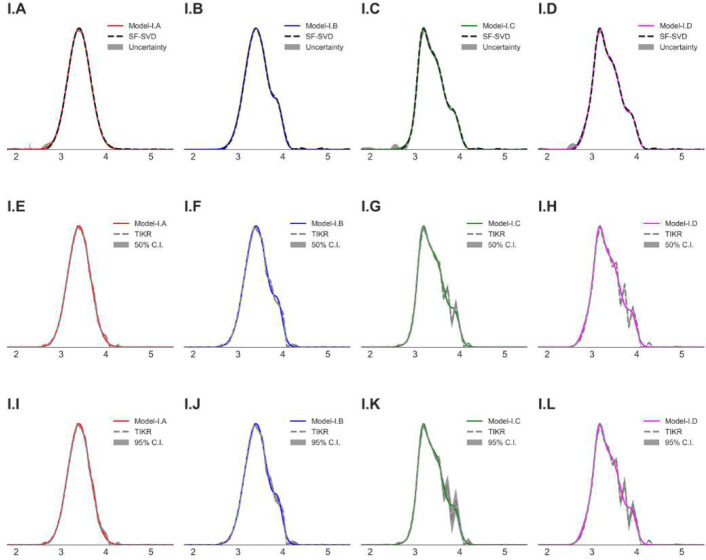
Model-I P(r) along with the reconstructed distance distributions by the (A-D) SF-SVD method and (E-L) the DEERLab Tikhonov regularization method (TIKR). The gray shaded regions represent the uncertainty for SF-SVD (A-D), the 50% (E-H) and the 95% (I-L) confidence intervals for TIKR.

**Fig. 5:**
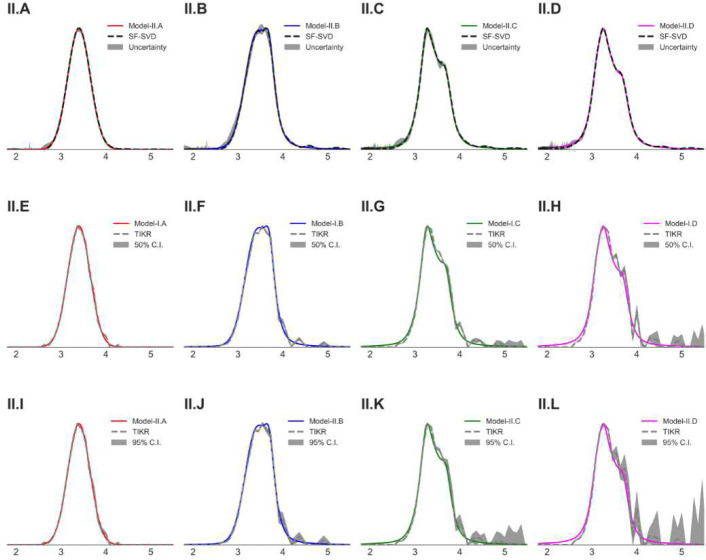
Model-II P(r) along with the reconstructed distance distributions by the (A-D) SF-SVD method and (E-H) the DEERLab Tikhonov regularization method (TIKR). The gray shaded regions represent the uncertainty for SF-SVD (A-D), the 50% (E-H) and 95% (I-L) confidence intervals for TIKR.

Reconstructions by the SF-SVD [16, 17] and the DEERLab TIKR method [24] were compared with the model distance distributions in Figures 4 and 5. While both methods captured major parts of the distance distribution patterns, the solutions varied significantly in some cases, e.g., (I.C-D) and (II.G-H) in Figure 4 and (II.B-D) and (II.F-H) in Figure 5. This raises considerable doubt over the true nature of the P(r) in such cases. The shaded (gray) region in those figures represents the uncertainty (SF-SVD), the 50% and 95% confidence intervals (DEERLab TIKR). For the SF-SVD solutions, the uncertainty is much less than those of the TIKR, especially for the multi-modal distributions. It is visible in both cases, but mainly for TIKR, that the uncertainty is greater in regions near the minor peak positions in the model P(r). More importantly, the 95% confidence interval for TIKR solutions, especially for model-II, shows large uncertainty associated with the solutions. At present, no cross validation method exists to confirm the existence of multi-modal distance distributions with one or more minor (or shoulder) peaks, which is necessary to improve the robustness of PDS analysis.

### 1.2. Proposed Method

Time-frequency analysis [25–30] is a reliable method to decouple a signal into its distinct constituent components by projecting it on the time-frequency plane. Short Time Fourier Transform (STFT) [25, 26, 29] is another strategy for such analysis, but a fixed window associated with STFT makes it unsuitable for separation of overlapping signal components. Wavelet transforms is a powerful method with great flexibility in time-frequency analysis and hence, it is extremely useful in extracting localized information from various types of signals [31, 32]. We propose the application of continuous wavelet transforms (CWT) in time-frequency analysis to (1) identify differences in P(r) for practically identical PDS signals and (2) confirm the existence of multimodal P(r).

## 2. Method

### 2.1. Wavelet Transform

A wavelet transform (WT) can simultaneously represent the time-frequency information for analysis through signal localization and is defined as [33–35]

(5) $$F\left(\tau ,\;s\right)=\frac{1}{\sqrt{\left|s\right|}}\int_{-\infty }^{+\infty }\;f\left(t\right)\psi^{*}\left(\frac{t\;-\;\tau }{s}\right)\;dt$$

where s is the inverse frequency (or frequency range) observing parameter (also called scale parameter), τ is the signal localization parameter (also called translation parameter), t represents the signal location, f(t) is the signal, F(τ,s) is the wavelet-transformed signal at a given signal localization and frequency, and $\psi^{*}\left(\frac{t-\tau }{s}\right)$ is the signal probing function obtained from a function called the “wavelet”, ψ(t). The functions ψ(t) and $\psi^{*}\left(\frac{t-\tau }{s}\right)$ are commonly referred as “mother” and “daughter” wavelet, respectively, because $\psi^{*}\left(\frac{t-\tau }{s}\right)$ is derived from ψ(t). $\psi^{*}(t)$ is the complex conjugate of ψ(t), which for a real function is the same, $\left(\psi^{*}(t)=\psi (t)\right)$.

The Fourier Transform (FT) can be considered as a special limiting case of the WT wherein $s\to (-i\omega {)}^{-1},\;\tau \to 0$, and $\psi \to e^{-i\omega t}$. Whereas a FT integrates out the time dependence, the WT is a function of both frequency, $s^{-1}$ and time, τ and thus can display correlations in the signal between them.

Unlike STFT, the WT employs a variable window width and a frequency parameter incorporated in the wavelet, that allows variation in both signal (e.g. time) and frequency. This informs about locations of a particular frequency in the signal domain as well as identifies all frequencies that are present at a particular signal location or interval. It results in analyzing a signal into different frequencies at different resolutions, allowing what is known as “multiresolution analysis”.

The wavelet-transformed signal F(τ,s) is represented in the signal domain at a frequency or frequency range, unlike the FT and STFT that represents signal just in the frequency domain. The location of data points in the wavelet domain is spatially correlated with the location of the signal domain. This reveals how a signal looks when observed from a specific frequency or frequency range.

The signal is reconstructed by inverse WT which is given as

(6) $$f\left(t\right)=\frac{1}{C_{\psi }^{2}}\int_{-\infty }^{+\infty }\int_{-\infty }^{+\infty }\frac{1}{s^{2}}F\left(\tau ,\;s\right)\psi \left(\frac{t\;-\;\tau }{s}\right)\;d\tau \;ds$$

where $C_{\psi }$ is admissibility constant which is written as

(7) $$C_{\psi }=\sqrt{2\pi \int_{-\infty }^{+\infty }\frac{|\Psi (\omega ){|}^{2}}{\left|\omega \right|}d\omega }<\infty$$

where Ψ(ω) is the FT of the wavelet function ψ(t). The constraint in Equation 7 implies that the wavelet function ψ(t) must also be oscillatory with zero mean i.e., $\int_{-\infty }^{+\infty }\;\psi (t)dt=0$

### 2.2. Discretized Continuous Wavelet Transform (CWT)

Similar to the Fourier Transform, the WT in Equation 5 is impractical for discrete data and a discretized version of CWT is used. For practical purposes, the translation parameter and the scale parameter are discretized as τ=a and s=b,a and b both being integers. The CWT of a discrete input signal is defined as

(8) $$C[a,\;b]=\frac{1}{\sqrt{b}}\sum_{m=0}^{p-1}f\left[t_{m}\right]\psi \left[\frac{t_{m}-a}{b}\right]$$

where, C[a,b] is the wavelet-transformed signal and $f\left[t_{m}\right]$ is the discrete input signal.

It should be noted that the discrete wavelet transform (DWT) is computationally more efficient than the CWT and applied more frequently. However, it is appropriate for extracting specific information from a signal. The CWT on the other hand is better suited for scanning all the time-frequency components in a signal for finer details and hence, it is better suited for this work.

### 2.3. CWT Time-Frequency Analysis in Python

Time-frequency analysis decouples a signal into its distinct constituent components by projecting it on the time-frequency plane. In this work, we used CWT time-frequency analysis of PDS signals and the Python script for that is as follows


## Python version 3.9 , 1 2
# **set** time domain
tm = 5 . 0 ; # **in** micro second
dt = 0 . 006 ; # **in** micro second
tx = numpy . arange (dt , tm + dt , dt) ;
# wavelet scale **to** use
import pywt
scales = np . **array** ( [ i **2 **for** i **in** range ( 1 , 1 9 ) ] ) ;
# **set** wavelet
waveletname = ‘ gaus2 ‘ ;
# calculate the cwt **of** the signal at given scales
[coef1 , freq1] = pywt . cwt (signal1 , scales , waveletname , dt )
# contour plots
import matplotlib . pyplot as plt
plt . contourf (tx , np . log (freq1) , abs (coef1) * * 0 . 5)


## 3. Results & Discussion

### 3.1. Time-Frequency Analysis of PDS Signals

**Fig. 6:**
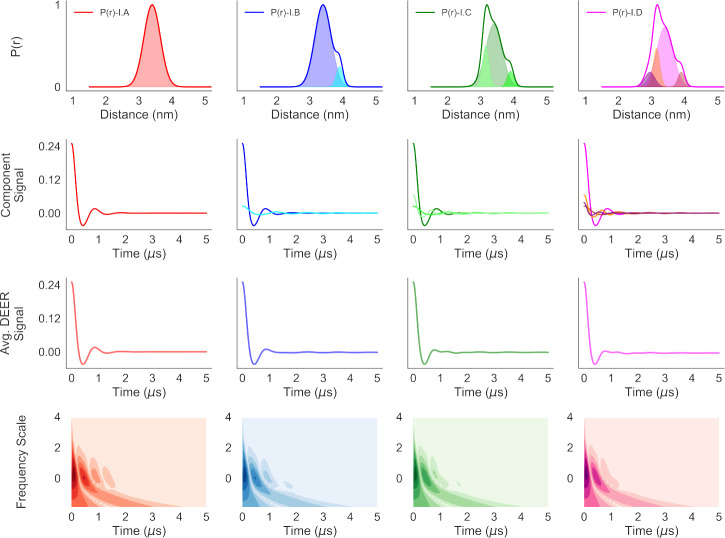
The similarity of the four DEER traces for model-I systems are reduced via the time-frequency analysis (bottom row) using CWT. The average P(r) (traces) along with the components (shaded distributions) are plotted in the top row. The resulting components of DEER signals and the average DEER traces are shown in the next two rows. The bottom row illustrates the time-frequency contour plots resulting from CWT of the signals using ‘Gaus2’ wavelet of the pywt Python library.

**Fig. 7:**
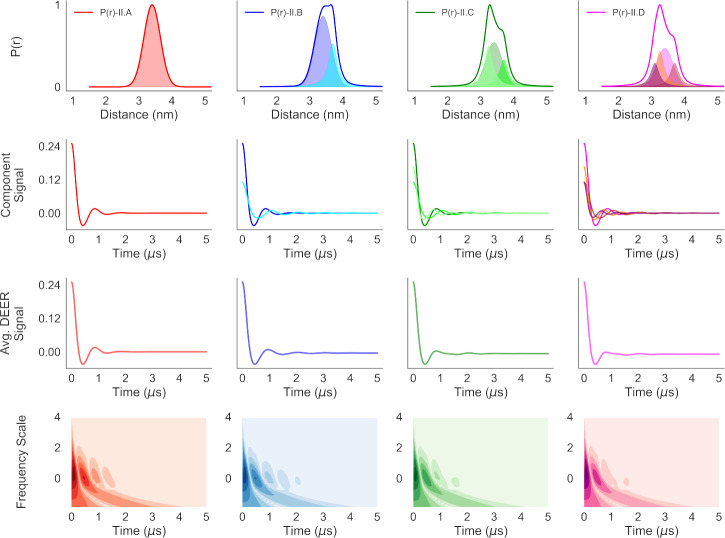
The similarity of the four DEER traces for model-II systems are reduced via the time-frequency analysis (bottom row) using CWT. The average P(r) (traces) along with the components (shaded distributions) are plotted in the top row. The resulting components of DEER signals and the average DEER traces are shown in the next two rows. The bottom row illustrates the time-frequency contour plots resulting from CWT of the signals using ‘Gaus2’ wavelet of the pywt Python library.

We calculated the CWT for the simulated DEER traces and plotted those in Figure 6 and Figure 7. The first and second rows in those figures show the component P(r) and the component DEER traces. Starting with a unimodal distribution (the far left column), minor components comprising Gaussian and/or Cauchy distributions were added to create the other P(r) and DEER signals. The resultant DEER signals were produced by adding the component DEER time-domain signals (third row) In Figure 6 and Figure 7, The resultant DEER signals are practically identical to those of the dominant component signals in the second rows of the figures because of the dominance of **P(r)-I.A** and **P**(**r**)-**II.A** in all the distance distributions in the two sets of model systems. In addition, summing of such signals with slightly different time-periods of oscillations causes destructive interference or smoothing of the features associated with the individual components. The bottom rows of those two figures show contour plots of the CWT of the DEER signals for different frequency scales vs. time. The Python script for calculation is given in Section 2. It can be noticed immediately that the CWT time-frequency contour plots in both Figure 6 and Figure 7 illustrate minor, but clearly visible differences, suggesting strong similarity among all the distance distributions, with minor, but detectable differences among all of them. Thus, the time-frequency analysis reveals significant information about different samples prior to the P(r) reconstruction process. On the other hand, such results for identical samples could indicate artifacts, reproducibility issues and inconsistency in sample preparation.

### 3.2. Time-Frequency Analysis and the Modality of the Distance Distributions

**Fig. 8:**
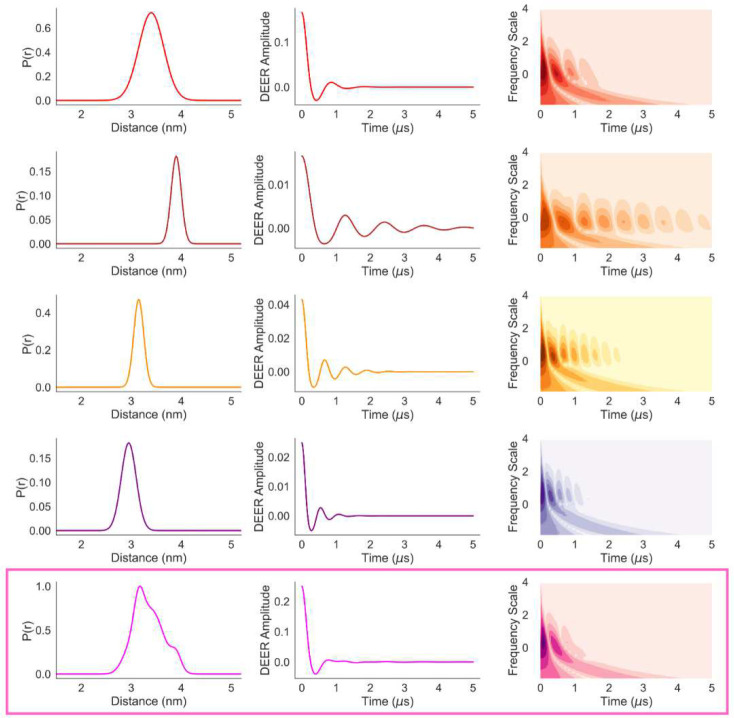
The four individual components of P(r)-I.D (left column), the corresponding DEER signals (middle column) and their CWT time-frequency plots (right column) are presented in the top four rows. The bottom row shows the summed P(r), DEER signal and its time-frequency plot (magenta rectangular box).

**Fig. 9:**
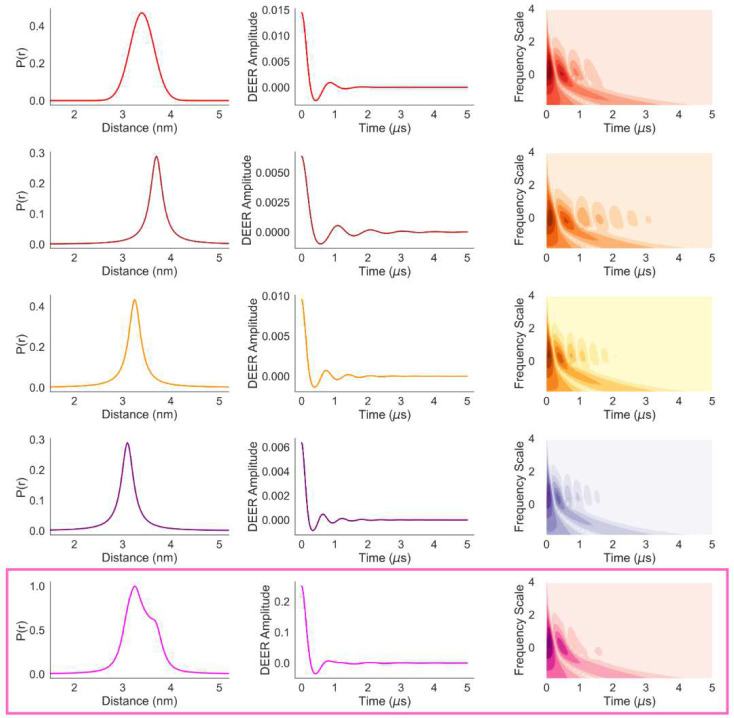
The individual components of P(r)-II.D (left column), the corresponding DEER signals (middle column) and their CWT time-frequency plots (right column) are presented in the top four rows. The bottom row shows the summed P(r), DEER signal and its time-frequency plot (magenta rectangular box).

For a qualitative analysis of the correlation between the differences in P(r) and the corresponding time-frequency contour plots, we have plotted the P(r) and DEER trace components along with their time-frequency plots for **P**(**r**)-**I.D** in Figure 8 (top four rows). The CWT time-frequency contour plots show that both the frequency and pattern along the time-domain varies with the modal distance of the distribution as well as its width. While the sum of the time-frequency plot shows close resemblance to the top row plot, indicating the dominance of the corresponding P(r) in the mixture, it also demonstrates clear differences. It can be seen that the time-frequency plots of the unimodal distributions have more prominent and periodic features compared to that of the summed signal. In multimodal and overlapping distance distributions, such periodic patterns tend to cancel out. Thus, a time-frequency plot with truncated features suggests the presence of such multimodal and overlapped distance distributions. The same observations are the case for model-II in Figure 9. The level of loss of the features is proportional to the number of closely spaced modal distances present in a distribution. Hence, it may be possible to train machine learning clustering algorithms against a large dataset of model distance distributions and their time-frequency patterns, which could then indicate the number of modal distances in a distribution.

### 3.3. Time-Frequency Analysis Using Different Wavelets

**Fig. 10:**
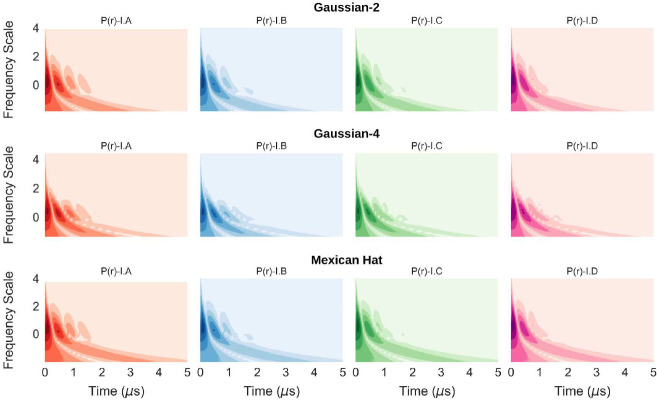
Time-frequency plots for model-I DEER time domain signals using three symmetric but different wavelets, namely: ‘Gaus2’ (top row), ‘Gaus4’ (middle row) and ‘Mexh’ (bottom row) wavelets of the pywt Python library.

In Figure 10, we repeated the time-frequency analysis for model-I DEER time domain signals using Gaussian-4 (‘Gaus4’) and Mexican Hat (‘Mexh’) wavelets and plotted the results along with that of the ‘Gaus2’ analysis, shown previously. Despite capturing different time-frequency sensitivity, core features of the CWT spectral pattern remain the same. Small variations between the CWT spectra reveal the sensitivity patterns for the different wavelets, but the core features remain the same. Hence, it can been seen that CWT spectra obtain for the dipolar signal is largely independent of the type of wavelet used, demonstrating the robustness of the analysis. We found that among the wavelet families available for CWT time-frequency analysis at present, the Gaussian family is the most suited for the analysis performed in this work. It is worth mentioning that other standard wavelets, such as Coiflet and Daubechies are not available for CWT analysis in Python or MATLAB at present. Therefore, we plan to develop new wavelets for deeper time-frequency analysis of PDS signals in the near future.

## 4. Conclusion

Through SDSL, spin labels are attached to specific domains of a protein and then application of PDS yields targeted structural information, i.e., distance distributions between the spin probes. The derivation of distance distributions is an ill-posed problem and in many cases, the results vary with the methods of reconstruction used in the analysis. In such cases, it is important to cross-validate the results and propose a solution with the least uncertainty. We illustrated in this work that continuous wavelet transform-based time-frequency analysis could be used for distingushing unimodal and multimodal distance distributions. We used eight model distance distributions and compared the solutions obtained from SF-SVD and the DEERLab Tikhonov regularization methods to illustrate the issue. The CWT time-frequency analysis reliably distinguishes between such very similar PDS signals, indicating the presence of unimodal vs. multimodal distance distributions. This method could be further developed for analysis of PDS signals to cross validate derived distance distributions and reduce the uncertainty associated with such analysis. In addition to model-free methods that generate distance distributions from dipolar signals, this method can also be potentially used with training-based P(r) reconstruction methods.

The future work will include development of Coiflet and Daubechies wavelets in CWT. Additionally, we plan to analyze a large dataset of PDS signals and employ appropriate machine learning tools to quantify the correlation between CWT time-frequency patterns and the number of peaks in the distance distributions.

## Data Availability

Data and the code used in this work have been stored in the public github repository (https://github.com/Signal-Science-Lab).

## Appendix A DEERLab Script

## P(r) reconstruction by Tikhonov regularization using DEERLab
import deerlab as dl
import numpy as np
import pandas as pd
import os
# filename without file format extension
tag = ‘signal2C’
# read experimental data
_mdir = “local repository”
_fname = tag+’.txt’
_data = np.loadtxt(os.path.join(_mdir,_fname), delimiter = ‘\t’)
t, Vexp = _data[:,0], _data[:,1]
# define model
tmin, tmax = 0.004, 2.
r = np.linspace(1,10,100)
expinfo = dl.ex_4pdeer(tau1=tmin, tau2=tmax, pathways=[1])
Vmodel = dl.dipolarmodel(t, r, Pmodel=None, Bmodel=None, experiment=expinfo)
# fit the model
compactness_penalty = dl.dipolarpenalty(None, r, ‘compactness’, ‘icc’)
result = dl.fit(Vmodel, Vexp, penalties=compactness_penalty)
# read the P(r)
pr = result.P
_norm = pr.max()
pr /= _norm
pci50 = result.PUncert.ci(50)
pci95 = result.PUncert.ci(95)

## References

[1] Hubbell W.L., Mchaourab H.S., Altenbach C., Lietzow M.A.: Watching proteins move using site-directed spin labeling. Structure 4(7), 779–783 (1996) 8805569 10.1016/s0969-2126(96)00085-8

[2] Hubbell W.L., Gross A., Langen R., Lietzow M.A.: Recent advances in site-directed spin labeling of proteins. Current Opinion in Structural Biology 8(5), 649–656(1998) 9818271 10.1016/s0959-440x(98)80158-9

[3] Hubbell W.L., Cafiso D.S., Altenbach C.: Identifying conformational changes with site-directed spin labeling. Nature Structural Biology 7(9), 735–739 (2000) 10966640 10.1038/78956

[4] Klug C.S., Feix J.B.: Methods and applications of site-directed spin labeling epr spectroscopy. Methods in cell biology 84, 617–658 (2008) 17964945 10.1016/S0091-679X(07)84020-9

[5] Borbat P., Costa-Filho A., Earle K., Moscicki J., Freed J.: Electron spin resonance in studies of membranes and proteins. Science 291(5502), 266–269 (2001) 11253218 10.1126/science.291.5502.266

[6] Borbat P.P., Mchaourab H.S., Freed J.H.: Protein structure determination using long-distance constraints from double-quantum coherence esr: Study of t4 lysozyme. Journal of the American Chemical Society 124(19), 5304–5314 (2002) 11996571 10.1021/ja020040y

[7] Borbat P.P., Freed J.H.: Measuring distances by pulsed dipolar ESR spectroscopy: Spin-labeled histidine kinases. Elsevier (2007) 10.1016/S0076-6879(07)23003-4 17609127

[8] Jeschke G.: Distance measurements in the nanometer range by pulse epr. ChemPhysChem 3(11), 927–932 (2002) 12503132 10.1002/1439-7641(20021115)3:11<927::AID-CPHC927>3.0.CO;2-Q

[9] Jeschke G.: Deer distance measurements on proteins. Annual Review of Physical Chemistry 63, 419–446 (2012) 10.1146/annurev-physchem-032511-143716 22404592

[10] Schweiger A., Jeschke G.: Principles of pulse electron paramagnetic resonance. Oxford university press (2001)

[11] Bertero M., Poggio T.A., Torre V.: Ill-posed problems in early vision. Proceedings of the IEEE 76(8), 869–889 (1988)

[12] Bertero M.: Linear inverse and iii-posed problems. In: Advances in Electronics and Electron Physics vol. 75, pp. 1–120. Elsevier, ??? (1989)

[13] Chiang Y.-W., Borbat P.P., Freed J.H.: The determination of pair distance distributions by pulsed esr using tikhonov regularization. Journal of Magnetic Resonance 172(2), 279–295 (2005) 15649755 10.1016/j.jmr.2004.10.012

[14] Chiang Y.-W., Borbat P.P., Freed J.H.: Maximum entropy: A complement to tikhonov regularization for determination of pair distance distributions by pulsed esr. Journal of Magnetic Resonance 177(2), 184–196 (2005) 16137901 10.1016/j.jmr.2005.07.021

[15] Brandon S., Beth A.H., Hustedt E.J.: The global analysis of deer data. Journal of Magnetic Resonance 218, 93–104 (2012) 22578560 10.1016/j.jmr.2012.03.006 PMC3608411

[16] Srivastava M., Freed J.H.: Singular value decomposition method to determine distance distributions in pulsed dipolar electron spin resonance. The Journal of Physical Chemistry Letters 8(22), 5648–5655 (2017) 29099190 10.1021/acs.jpclett.7b02379 PMC5708871

[17] Srivastava M., Freed J.H.: Singular value decomposition method to determine distance distributions in pulsed dipolar electron spin resonance: Ii. estimating uncertainty. The Journal of Physical Chemistry A 123(1), 359–370 (2018) 30525624 10.1021/acs.jpca.8b07673 PMC6372347

[18] Edwards T.H., Stoll S.: Optimal tikhonov regularization for deer spectroscopy. Journal of Magnetic Resonance 288, 58–68 (2018) 29414064 10.1016/j.jmr.2018.01.021 PMC5840305

[19] Matveeva A.G., Yushkova Y.V., Morozov S.V., Grygor’ev I.A., Dzuba S.A.: Multi-gaussian monte carlo analysis of peldor data in the frequency domain. Zeitschrift für Physikalische Chemie 231(3), 671–688 (2017)

[20] Timofeev I.O., Krumkacheva O.A., Fedin M.V., Karpova G.G., Bagryanskaya E.G.: Refining spin-spin distance distributions in complex biological systems using multi-gaussian monte carlo analysis. Applied Magnetic Resonance 49, 265–276(2018)

[21] Sweger S.R., Pribitzer S., Stoll S.: Bayesian probabilistic analysis of deer spectroscopy data using parametric distance distribution models. The Journal of Physical Chemistry A 124(30), 6193–6202 (2020) 32614584 10.1021/acs.jpca.0c05026 PMC7846514

[22] Worswick S.G., Spencer J.A., Jeschke G., Kuprov I.: Deep neural network processing of deer data. Science Advances 4(8), 5218 (2018) 10.1126/sciadv.aat5218 PMC6108566 30151430

[23] Keeley J., Choudhury T., Galazzo L., Bordignon E., Feintuch A., Goldfarb D., Russell H., Taylor M.J., Lovett J.E., Eggeling A., : Neural networks in pulsed dipolar spectroscopy: A practical guide. Journal of Magnetic Resonance 338, 107186(2022) 35344921 10.1016/j.jmr.2022.107186

[24] Ibáñez L.F., Jeschke G., Stoll S.: Deerlab: A comprehensive software package for analyzing dipolar electron paramagnetic resonance spectroscopy data. Magnetic Resonance (Gottingen, Germany) 1(2), 209 (2020) 34568875 10.5194/mr-1-209-2020 PMC8462493

[25] Langmead C.J., Donald B.R.: Extracting structural information using time-frequency analysis of protein nmr data. In: Proceedings of the Fifth Annual International Conference on Computational Biology, pp. 164–175 (2001)

[26] Daubechies I.: The wavelet transform, time-frequency localization and signal analysis. IEEE Transactions on Information Theory 36(5), 961–1005 (1990)

[27] Popiński W.: Wavelet transform for time-frequency representation and filtration of discrete signals. Applicationes Mathematicae 23(4), 433–448 (1996)

[28] Constable R., Thornhill R.: Using the discrete wavelet transform for time-frequency analysis of the surface emg signal. Biomedical Sciences Instrumentation 29, 121–127 (1993) 8329582

[29] Brotherton T., Pollard T., Barton R., Krieger A., Marple L.: Application of time-frequency and time-scale analysis to underwater acoustic transients. In: [1992] Proceedings of the IEEE-SP International Symposium on Time-Frequency and Time-Scale Analysis, pp. 513–516 (1992). IEEE

[30] Qin L., He B.: A wavelet-based time–frequency analysis approach for classification of motor imagery for brain–computer interface applications. Journal of Neural Engineering 2(4), 65 (2005) 16317229 10.1088/1741-2560/2/4/001

[31] Roy A.S., Srivastava M.: Hyperfine decoupling of esr spectra using wavelet transform. Magnetochemistry 8(3), 32 (2022) 37475982 10.3390/magnetochemistry8030032 PMC10357921

[32] Sinha Roy A., Srivastava M.: Analysis of small-molecule mixtures by super-resolved 1h nmr spectroscopy. The Journal of Physical Chemistry A 126(48), 9108–9113 (2022) 36413171 10.1021/acs.jpca.2c06858 PMC10228708

[33] Daubechies I.: Ten lectures on wavelets. SIAM (1992)

[34] Mallat S.: A wavelet tour of signal processing. Elsevier (1999)

[35] Addison P.S.: The illustrated wavelet transform handbook: Introductory theory and applications in science, engineering, medicine and finance. CRC Press (2017)

